# *FBXL4*-Related Mitochondrial DNA Depletion Syndrome 13 (MTDPS13): A Case Report With a Comprehensive Mutation Review

**DOI:** 10.3389/fgene.2019.00039

**Published:** 2019-02-05

**Authors:** Rami A. Ballout, Chadi Al Alam, Penelope E. Bonnen, Martina Huemer, Ayman W. El-Hattab, Rolla Shbarou

**Affiliations:** ^1^Faculty of Medicine, American University of Beirut, Beirut, Lebanon; ^2^Division of Pediatric Neurology, Department of Pediatrics, American University of Beirut Medical Center, Beirut, Lebanon; ^3^Department of Molecular and Human Genetics, Baylor College of Medicine, Houston, TX, United States; ^4^Department of Pediatrics, Landeskrankenhaus Bregenz, Bregenz, Austria; ^5^Division of Metabolism, University Children's Hospital Zürich, Zurich, Switzerland; ^6^Genetics Clinic, KidsHeart Medical Center, Dubai, United Arab Emirates

**Keywords:** mitochondria, mitochondrial diseases, mitochondrial fusion, mitochondrial DNA (mtDNA), FBXL4, pediatric genetics, rare diseases, genetic mutations

## Abstract

Mitochondrial DNA depletion syndromes (MTDPS) are a group of rare genetic disorders caused by defects in multiple genes involved in mitochondrial DNA (mtDNA) maintenance. Among those, *FBXL4* mutations result in the encephalomyopathic mtDNA depletion syndrome 13 (MTDPS13; OMIM #615471), which commonly presents as a combination of failure to thrive, neurodevelopmental delays, encephalopathy, hypotonia, and persistent lactic acidosis. We report here the case of a Lebanese infant presenting to us with profound neurodevelopmental delays, generalized hypotonia, facial dysmorphic features, and extreme emaciation. Whole-exome sequencing (WES) showed the girl as having MTDPS13 with an underlying *FBXL4* missense mutation that has been previously reported only twice in unrelated individuals (c.1303C > T). Comprehensive literature search marked our patient as being the 94th case of MTDPS13 reported to date worldwide, and the first from Lebanon. We include at the end of this report a comprehensive mutation review table of all the pathological *FBXL4* mutations reported in the literature, using it to highlight, for the first time, a possible founder effect of Arab origins to the disorder, being most prevalent in patients of Arab descent as shown in our mutation table. Finally, we provide a direct comparison of the disorder's clinical manifestations across two unrelated patients harboring the same disease-causing mutation as our patient, emphasizing the remarkable variability in genotype-to-phenotype correlation characteristic of the disease.

## Introduction

Mitochondrial DNA (mtDNA) synthesis is an intricate and complex process requiring specific stoichiometric proportions of replicative enzymes and a tightly regulated pool of mitochondrial nucleotides (El-Hattab et al., 2017b). While the latter is maintained through the concerted actions of carrier-mediated transport of nucleotides from the cytosol into the mitochondria and a specialized nucleotide salvage pathway within mitochondria, the former obligates that mitochondria fuse and exchange their contents, including replisome components (Chen et al., 2010; El-Hattab et al., 2017b; Viscomi and Zeviani, 2017). Thus, mutations affecting genes integral to any of these processes, including mitochondrial fusion, can result in impaired mtDNA synthesis and subsequently, mtDNA depletion (El-Hattab et al., 2017b; Viscomi and Zeviani, 2017).

Among the proteins recently reported as mutated in a subset of patients with mtDNA depletion syndromes is the F-box and leucine-rich repeat 4 protein (FBXL4) (Bonnen et al., 2013; Huemer et al., 2015; Almannai et al., 2017; Dai et al., 2017), an orphan member of the F-box family of proteins that are key components of the Skp1-Cullin-F-box (SCF) ubiquitin ligase complex (Bai et al., 1996), and thus, play a role in ubiquitination (Cenciarelli et al., 1999; Winston et al., 1999). The protein has also been shown to possess a mitochondrial-translocation signal that targets its trafficking into the inter-membrane space of mitochondria (Bonnen et al., 2013; Gai et al., 2013). Despite previous studies showing a reduced mtDNA content, lower steady-state levels of the mtDNA-encoded respiratory chain enzyme subunits, and a fragmented mitochondrial network with an overall reduced mitochondrial mass in FBXL4-deficient cells (Bonnen et al., 2013; Gai et al., 2013; Huemer et al., 2015), the precise role of FBXL4 remains unknown.

Encephalomyopathic mtDNA depletion syndrome 13 (MTDPS13; OMIM # 615471) is an extremely rare autosomal recessive disorder arising from bi-allelic mutations in the *FBXL4* gene (MIM 605654). Clinically, MTDPS13 typically presents with failure to thrive, remarkable neurodevelopmental delays, encephalopathy, cerebral atrophy, generalized hypotonia, and persistent lactic acidosis (Bonnen et al., 2013; Gai et al., 2013; Huemer et al., 2015; Almannai et al., 2017). Additionally, biochemical and molecular testing on samples obtained from MTDPS13-affected subjects show mtDNA depletion, abnormal mitochondrial morphology, and electron transfer chain defects (Bonnen et al., 2013; Gai et al., 2013; Huemer et al., 2015). To our best knowledge, only 93 cases of FBXL4 deficiency have been reported to date in the literature (Shamseldin et al., 2012; Bonnen et al., 2013; Gai et al., 2013; Huemer et al., 2015; Wortmann et al., 2015; Antoun et al., 2016; Barøy et al., 2016; Morton et al., 2016; Pronicka et al., 2016; van Rij et al., 2016; Dai et al., 2017; El-Hattab et al., 2017a), with no prior attempts of elucidating any potential ethnic correlations.

Thus, we decided to report herein the case of a 9 month-old girl of Lebanese-Arab origins who presented to us with pervasive neurodevelopmental delays and generalized hypotonia, first noted by her parents at the age of 3 months. After a series of investigations, the girl was diagnosed with *FBXL4*-related MTDPS13, resulting from a homozygous nonsense mutation [NM_012160.4 (FBXL4): c.1303C>T (p.Arg435^*^)] acquired from her carrier parents. Her mutation has only been reported twice within the literature before (OMIM: 605654.0002) (Bonnen et al., 2013; Huemer et al., 2015), yet displays a vast genotype-phenotype variability when comparing her to the two other cases. We also provide a comprehensive summary of all FBXL4-deficient cases reported to date in the literature, including our case, to act as a future mutation reference table for updates and association studies.

## Background

A 9 month-old girl of Lebanese-Arab origins was brought to us by her parents for evaluation of her remarkably delayed acquisition of age-appropriate neurodevelopmental milestones, along with generalized hypotonia, first noticed at the age of 3 months. The parents are first-degree cousins with an extensive history of consanguinity in either side of the family. However, both family histories are negative for any neurological disorders or developmental abnormalities. The proband was born at term via an elective C-section, after a non-remarkable prenatal history. However, her birth weight, height, and occiput-frontal circumference were all at the 5th percentiles (2.4 kg, 46 and 33 cm, respectively).

Newborn screening was non-revealing and she had an uneventful postnatal course. She was exclusively breast-fed until the age of 3 months when she contracted pneumonia that necessitated and intravenous antibiotics for 10 days. However, she gradually recovered afterwards with no further complications. Nonetheless, since discharge and up until the age of 6 months, her parents had regularly noticed a persistent head drop that failed to catch up by 6 months of age, along with an inability to roll-over or sit supported by that time. However, the daughter was never evaluated by a pediatrician since birth.

On inspection, the girl exhibits profound facial dysmorphic features, including a bossing forehead, supraorbital ridge protuberance, long and dark eyelashes, thick and fuzzy eyebrows with remarkable hirsutism, and a smooth and long philtrum.

Physical examination revealed a distended round abdomen with an otherwise paradoxical emaciated habitus. No signs of dehydration were noted, and the mother confirmed adequate oral intake. Her weight was 3.2 kg (−6 SD below the mean) and her height was 60 cm (−3.5 SD below the mean). She exhibited severe microcephaly with a head circumference of 39 cm (−4 SD below the mean).

History excluded dysphagia, feeding difficulties, and any gastrointestinal manifestations that can explain her reduced weight. However, they reported a poor development in her mastication skills, necessitating her maintenance on a mashed diet to avoid any choking or aspiration.

Neurologically, the girl was overall hypotonic with diffuse muscle wasting, yet no dystonic features. Her Denver developmental scale II (DDS-II) assessment confirmed her inability to roll-over or sit with support as reported by the mother, and additionally highlighted deficits in pointing at or reaching out to objects of interest. Of note however, age-appropriate social behavior and skills were normal, and she was able to recognize the smiles of her parents and coo to them. She displayed no strabismus or nystagmus, and tracked movement normally. Thus, her developmental age at the time was estimated as being 3–4 months, profoundly delayed relative to her chronological age (i.e., 9 months).

Basic laboratory studies showed severe anemia (hemoglobin 6.82g/dL [*N*: 11.1–14.1), mild leukopenia (4,710 white blood cells/mm^3^ [*N*: 5,000–10,000]), thrombocytosis (617,000 platelets/uL [*N*: 150,000–400,000]), hypoalbuminemia (29 [*N*: 36–53]), normal blood urea nitrogen level (15 mg/dL [*N*: 8–25]), mild lactic acidosis (3.5 mmol/L [*N*: < 2.2]), and markedly elevated C-reactive peptide level (CRP; 251 mg/L [*N*: < 2.5]). Of interest, her labs showed no hyperammonemia (28 umol/L [*N*: < 50]) or ketosis. Urinalysis and chest X-ray were normal, and her plasma and urine amino and organic acid levels were within reference range.

Ophthalmological evaluation of extraocular movements and gaze, as well as funduscopic (retinal) examination were all normal. Otoacoustic emission testing, echocardiography, and abdominal ultrasound were normal as well. However, brain magnetic resonance imaging (MRI) showed bilateral fronto-temporal atrophy and ventricular enlargement, in the absence of hydrocephalus, intracranial calcifications, or any hypo-intense lesions.

At this point, we sought whole-exome sequencing (WES) for the trio, only to discover that the proband is carrying a homozygous non-sense mutation in *FBXL4* [NM_012160.4: c.1303C>T (p.Arg435^*^)], diagnosing her consequently with *FBXL4*-related MTDPS13. The parents were both carriers of that mutation.

However, our patient was lost to follow up after her diagnosis at the age of 9 months, despite several attempts to have her parents return for follow up. However, we did reach out to the family afterwards to check on the girl's status at the time, to learn that she was 2 years and 8 months old back then, with a persistence of her inability to walk or talk. At that instance, we obtained parental written consent to have their daughter's rare case reported.

## Discussion

To our best knowledge, our patient constitutes the 94th case of *FBXL4*-related encephalomyopathic MTDPS13 reported to date in the literature (Supplementary Table; Shamseldin et al., 2012; Bonnen et al., 2013; Gai et al., 2013; Huemer et al., 2015; Wortmann et al., 2015; Antoun et al., 2016; Barøy et al., 2016; Morton et al., 2016; Pronicka et al., 2016; van Rij et al., 2016; Dai et al., 2017; El-Hattab et al., 2017a). Despite exhibiting several of the “typical” manifestations reported previously in MTDPS13 such as hypotonia, encephalopathy with brain atrophy, lactic acidosis, and stunted growth (Bonnen et al., 2013; Gai et al., 2013; Huemer et al., 2015; Almannai et al., 2017; El-Hattab et al., 2017a), our patient also had two rather “unusual” findings worth highlighting: severe anemia, and a normal blood ammonia level (i.e., absence of hyperammonemia) (Almannai et al., 2017). Nonetheless, the absence of feeding problems, discernible GI (normal and regular bowel movements, negative stool culture, and normal ultrasound) or liver pathology (no jaundice, normal liver enzymes, and only borderline low albumin), and lack of ketones in urine, along with a normal MCV, rule out nutritional deficiency or underfeeding as the likely etiologies of her severe anemia. In this case, one may argue that her anemia is just another example of the genotype-phenotype variability characteristic of the disorder, where in her case there is an involvement of her bone marrow stem cell population in the disease, taking into consideration her other aforementioned hematological abnormalities (leukopenia and thrombocytosis).This however remains only a hypothetical claim, with the precise reason for her severe anemia remaining unclear.

Her mutation [c.1303C>T (p.Arg435^*^)] has been previously identified elsewhere only twice, in two unrelated individuals in 2013 (Bonnen et al., 2013) and 2015 (Huemer et al., 2015). Of interest, when we sought the individual patient data from the lead authors of those two studies, we discovered that these two other patients sharing our patient's mutation were also born to consanguineous Arab parents.

To date, a total of 47 pathogenic mutations have been identified in the *FBXL4* locus (Table 1), the majority of which are missense (24/47; 51%) and non-sense mutations (10/47; 21%). Only a minute fraction are actually frameshift (7/47; 15%) or splice-site mutations (6/47; 13%).

**Table 1:** List of all pathological FBXL4 genotypes reported to date in the literature.

**Allele 1**	**Allele 2**	**Type of mutation(s) (Allele 1/ Allele 2)**	**Zygosity**	**Study reporting it**	**Number of patients**
c.1304G>T (p.Arg435Leu)	c.1304G>T (p.Arg435Leu)	Missense/Missense	Homozygous	Huemer et al., 2015	1
c.513-1G>A	c.513-1G>A	Splice-site/Splice-site	Homozygous		1
c.1361A>C (p.Gln454Pro)	c.1361A>C (p.Gln454Pro)	Missense/Missense	Homozygous		1
c.662A>T (p.Asp221Val)	c.662A>T (p.Asp221Val)	Missense/Missense	Homozygous		1
c.1304G>A (p.Arg435Gln)	c.1232G>A (p.Cys411Tyr)	Missense/Missense	Compound heterozygous		2
c.661G>C; (p.Asp221His)	c.1641_1642delTG (p.Cys547fs)	Missense/frameshift	Compound heterozygous		1
c.1772A>G (p.Asp591Gly)	c.1772A>G (p.Asp591Gly)	Missense/Missense	Homozygous		1
c.1067delG (p.Gly356Alafs^*^15)	c.1067delG (p.Gly356Alafs^*^15)	Frameshift/frameshift	Homozygous		1
c.1232G>A (p.Cys411Tyr)	c.1232G>A (p.Cys411Tyr)	Missense/Missense	Homozygous		1
c.1444C>T (p.Arg482Trp)	c.1546_1563del (p.Pro516_Ser521del)	Missense/frameshift	Compound heterozygous		1
c.445G>A (p.Gly149Arg)	c.445G>A (p.Gly149Arg)	Missense/Missense	Homozygous		1
c.1703G>C (p.Gly568Ala)	c.316C>T (p.Gln106^*^)	Missense/Non-sense	Compound heterozygous	Huemer et al., 2015 El-Hattab et al., 2017a	1 2
c.1555C>T (p.Gln519^*^)	c.1555C>T (p.Gln519^*^)	Non-sense/Non-sense	Homozygous	Bonnen et al., 2013; Huemer et al., 2015; El-Hattab et al., 2017a	5 3 2
c.1303C>T (p.Arg435^*^)	c.1303C>T (p.Arg435^*^)	Non-sense/Non-sense	Homozygous	Bonnen et al., 2013; Huemer et al., 2015 Our patient	5 1 1
c.1703G>C (p.Gly568Ala)	c.1703G>C (p.Gly568Ala)	Missense/Missense	Homozygous	Bonnen et al., 2013 Shamseldin et al., 2012; Dai et al., 2017; El-Hattab et al., 2017a	1 1 1 2
c.219T>A (p.Tyr73^*^)	c.219T>A (p.Tyr73^*^)	Non-sense/Non-sense	Homozygous	Dai et al., 2017; El-Hattab et al., 2017a	1 1
c.1687C>T (p.Gln563^*^)	c.1622C>T (p.Thr541Ile)	Non-sense/missense	Compound heterozygous	Dai et al., 2017	1
c.1838T>A (p.Val613Glu)	c.1389+3_+6delAAGT	Missense/splice-site	Compound heterozygous		1
c.64C>T (p.Arg22^*^)	c.1444C>T (p.Arg482Trp)	Non-sense/missense	Compound heterozygous		1
c.273_277del (p.Phe91fs)	c.273_277del (p.Phe91fs)	Frameshift/frameshift	Homozygous		1
c.1641_1642delTG (p.Cys547fs)	c.1232G>A (p.Cys411Tyr)	Frameshift/missense	Compound heterozygous		1
c.64C>T (p.Arg22^*^)	c.1641_1642delTG (p.Cys547fs)	Non-sense/frameshift	Compound heterozygous		1
c.1641_1642delTG (p.Cys547fs)	c.1641_1642delTG (p.Cys547fs)	Frameshift/frameshift	Homozygous	Antoun et al., 2016; Dai et al., 2017	1 1
c.1442T>C (p.Leu481Pro)	c.1442T>C (p.Leu481Pro)	Missense/Missense	Homozygous	Barøy et al., 2016	1
c.1586C>A (p.Ala529Glu)	c.1790A>C (p.Gln597Pro)	Missense/Missense	Compound heterozygous	Morton et al., 2016	2
c.292C>T (p.Arg98^*^)	c.1303C>T (p.Arg435^*^)	Non-sense/Non-sense	Compound heterozygous	van Rij et al., 2016	1
c.858+1G>T	c.585+5G>C	Splice-site/Splice-site	Compound heterozygous	Pronicka et al., 2016	1
c.1303C>T (p.Arg435^*^)	c.64C>T (p.Arg22^*^)	Non-sense/Non-sense	Compound heterozygous		1
c.64C>T (p.Arg22^*^)	c.64C>T (p.Arg22^*^)	Non-sense/Non-sense	Homozygous		1
c.1411G>A (p.Ala471Thr)	c.1790A>C (p.Gln597Pro)	Missense/Missense	Compound heterozygous	Wortmann et al., 2015	2
c.1694A>G (p.Asp565Gly)	c.1694A>G (p.Asp565Gly)	Missense/Missense	Homozygous	Gai et al., 2013; El-Hattab et al., 2017a	1 1
c.1444C>T (p.Arg482Trp)	c.1444C>T (p.Arg482Trp)	Missense/Missense	Homozygous	Gai et al., 2013	3
c.1652T>A (p.Ile551Asn)	c.1652T>A (p.Ile551Asn)	Missense/Missense	Homozygous		1
c.1790A>C (p.Gln597Pro)	c.1067delG (p.Gly356fs)	Missense/frameshift	Compound heterozygous		1
c.614T>C (p.Ile205Thr)	c.106A>T (p.Arg36^*^)	Missense/non-sense	Compound heterozygous		1
c.1229C>T (p.Ser410Phe)	c.1229C>T (p.Ser410Phe)	Missense/Missense	Homozygous		1
c.616C>T (p.Arg206^*^)	c.616C>T (p.Arg206^*^)	Non-sense/Non-sense	Homozygous	El-Hattab et al., 2017a	2
c.1750T>C (p.Cys584Arg)	c.1750T>C (p.Cys584Arg)	Missense/Missense	Homozygous		1
c.292C>T (p.Arg98^*^)	c.292C>T (p.Arg98^*^)	Non-sense/Non-sense	Homozygous		2
c.1698A>G (p.Ile566Met)	c.1698A>G (p.Ile566Met)	Missense/Missense	Homozygous		14
c.1317G>A	c.1317G>A	Splice-site/Splice-site	Homozygous		1
c.1648_1649delGA (p.Asp550Hisfs^*^2)	c.1648_1649delGA (p.Asp550Hisfs^*^2)	Frameshift/Frameshift	Homozygous		2
c.1641_1642delTG (p.Cys547fs)	c.419T>C (p.Val140Ala)	Frameshift/missense	Compound heterozygous		1
c.419T>C (p.Val140Ala)	c.419T>C (p.Val140Ala)	Missense/Missense	Homozygous		1
c.1210C>T (p.Gln404^*^)	c.1210C>T (p.Gln404^*^)	Non-sense/Non-sense	Homozygous		1
c.1641_1642delTG (p.Cys547fs)	c.618_621dupACTG (p.Glu208Thrfs^*^5)	Frameshift/frameshift	Homozygous		1
c.1540T>G (p.Trp514Gly)	c.1540T>G (p.Trp514Gly)	Missense/Missense	Homozygous		1
c.326delG; (p.Ser109Metfs^*^35)	c.1622C>T (p.Tyr541Ile)	Frameshift/missense	Compound heterozygous		1
exons 2, 3, 4del	exons 2, 3, 4del	Exonic deletion/Exonic deletion	Homozygous		1
				Total	94

Of noteworthy however, among all *FBXL4* genotypes reported to date in the literature (Table 1; *n* = 49), four genotypes particularly appear to be the most prevalent, affecting more than one-third of all FBXL4 deficiency patients reported to date (36 out of 94 i.e., 38%). Among those, the homozygous [c.1698A>G (p.Ile566Met)] genotype arising from a missense mutation that has only been recently identified by El Hattab et al. in 14 of their 37 patients, ranks highest in prevalence, affecting nearly 15% (14/94) of all FBXL4 deficiency patients reported to date (El-Hattab et al., 2017a). In contrast, the second and third most common genotypes were both the result of homozygous non-sense (i.e., null) mutations ([c.1555C>T (p. Gln519^*^)] and [c.1303C>T (p. Arg435^*^)] ,respectively), detected in 11% (10/94) and 7% (7/94), respectively, of all listed FBXL4 deficiency patients. Finally, the fourth most common genotype apparently is the result of a homozygous missense mutation [c.1703G>C (p.Gly568Ala)] that has been seen in 5 of the 94 patients identified to date (i.e., 5%).

Of further interest and through reviewing patient demographic data, the four aforementioned genotypes affecting more than a third of all identified FBXL4 deficiency patients were all actually identified in Arab patients exclusively, suggesting a possibly higher prevalence of the condition among Arabs compared to other ethnic groups. However, the reasons for this remain unknown at the moment. High consanguinity rates characteristic of this ethnic group may partially but not completely account for this observation (Inhorn et al., 2009; Tadmouri et al., 2009), and/or alternatively, Arabs may have higher carrier-status rates of *FBXL4* mutations compared to other ethnicities, similar to the higher Huntington's disease (i.e., CAG triplet repeat expansion) carrier rates among Europeans compared to other populations as an analogy (Orth et al., 2010). Alternatively, a founder's effect for the disorder among Arabic populations can be a reasonable argument when considering that the disorder's four predominant genotypes, accounting for more than one-third of the patients reported thus far with the disorder globally, were seen exclusively in patients of Arab descent. However, the disorder itself as summarized in Table 1 has been reported in various ethnic groups as well, though not as apparently predominant as in Arabs. These explanations however remain only hypothetical suggestions, with the true reasons behind this observation being unknown at the moment, yet a worthwhile target of future investigation.

Despite her poor neurodevelopmental outcome, our patient was still alive when we last heard from the family at the age of 2 years and 8 months, unlike the patient reported by Bonnen et al. (boy; S2) who had the same genotype yet passed away at the age of 4 months (Bonnen et al., 2013). In fact, the latter had three siblings and one cousin with the same genotype, presenting at a much earlier age compared to our patient, with all of them passing away shortly after birth (Bonnen et al., 2013). However, no information is available on the fate of the other patient carrying the same mutation and reported by Huemer et al. (patient 13), due to loss of follow up (Huemer et al., 2015).

Table 2 compares the presentations of all three patients mentioned above, highlighting the broad phenotypic spectrum of the disorder among patients with identical genotypes. In particular, the age at onset of symptoms was remarkably different, ranging from immediately postpartum (patient 13) (Huemer et al., 2015) to 3 months of age in the case of our patient, and an intermediary age of symptom onset for the third patient (S2) who first developed symptoms within the first month after birth (Bonnen et al., 2013). Moreover, our patient did not have cardiomyopathy and only had a mildly elevated lactic acid level (3.5 mmol/L) compared to the two other patients (S2 and patient 13) who both had discernible cardiomyopathy and remarkably high (and equal) levels of lactic acidosis (18 mmol/L). This possibly suggests an “attenuated” version of the disorder in our patient compared to the two other patients reported elsewhere, given her longer survival and lower comorbidities. Interestingly, while both, our patient and patient 13 had no cataracts at birth, S2 had bilateral cataracts with optic atrophy. Additionally, while our patient and S2 both displayed the disorder's “typical” facial dysmorphic features, patient 13 showed no facial dysmorphism. This clearly demonstrates the non-true-breeding nature of *FBXL4*-related MTDPS13, which can clearly present with a broad array of phenotypic characteristics, even amongst patients with the same genotypes.

**Table 2:** A direct comparison of our patient's manifestations to those of the two other patients reported elsewhere with an identical *FBXL4* genotype.

	**Our patient**	**Bonnen et al. (2013) (S2)**	**Huemer et al. (2015) (Patient 13)^†^**
Sex	Female	Male	Male
Age of symptom onset	3 months	Before 1 month	At birth (day 0)
Age at presentation	9 months	1 month	1 day
Birthweight (kg)	2.4	2.3	Unknown
Hypotonia	Severe	Severe	Severe
Cataracts	Absent	Present, with optic atrophy	Absent
Cardiac anomalies	None	Cardiomyopathy	Cardiomyopathy
Brain MRI findings	Bilateral temporal and frontal atrophy with non-hydrocephalus ventriculomegaly.	Prominence of the Cisterna Magna, with an abnormal appearance of the dorsal brainstem and posterior limb of the internal capsule.	Cerebellar hypoplasia
Lactic acid levels (mmol/L)	3.5	18	18
Ammonia levels	Non-elevated	Non-elevated	Unknown
Age at death	Lost to follow-up ∧	4 months	Lost to follow-up

† *This boy did not have any facial dysmorphic features*.

∧ *Patient was still alive at the age of 2 years and 8 months, after which she was lost to follow-up*.

Finally, based on the biochemical and cellular alterations seen with pathological *FBXL4* mutations, FBXL4 protein is now believed to play a key role in oxidative phosphorylation and mitochondrial dynamics, primarily mitochondrial fusion, a process key for mtDNA maintenance (Bonnen et al., 2013; Gai et al., 2013; Huemer et al., 2015; El-Hattab et al., 2017a). Additionally, with recent findings that somatic *FBXL4* mutations increase the likelihood of prostate cancer metastasis to bone (Stankiewicz et al., 2017), and modify the circadian GABA-ergic cyclical alteration in lateral ventral nuclei of drosophila (Li et al., 2017), suggest not only a potential role for FBXL4 in cell migration, but possibly in the sleep-wake cycle as well, respectively. Nonetheless, the precise function of FBXL4 remains poorly defined and a subject of further research.

## Concluding Remarks

We hope our case sheds light on the higher prevalence of *FBXL4*-related MTDPS13 among patients of Arabic ethnicity, while highlighting the disease' characteristically remarkable genotype-phenotype variability nature. We hope that this report also serves to raise awareness among pediatric neurologists and medical geneticists of the rare yet disabling nature of mtDNA maintenance defects such as *FBXL4*-deficiencies, and therefore, the importance of pursuing further research in the field to better characterize the exact function(s) of FBXL4. Such an understanding would allow better identification of possible targets for future therapeutic intervention that would aim at completely replacing the defective protein, or alternatively, augmenting any residual activity it may possess.

## Ethics Statement

This study was exempt from ethical approval procedures being a case report of a single patient whose parents have voluntarily provided oral consent to participate in the study, and written consent to have her case published. As per the Institutional Review Board (IRB) of the American University of Beirut, no pre-approval is required for the publication of a case report of a single patient.

## Author Contributions

RB was the third-year medical student interviewing the patient's parents during their first visit and performing the initial physical examination of the patient that was replicated by CA (the fellow in pediatric neurology) for confirmation. RB and CA finalized the case with RS, the pediatric neurologist, who then requested the performed tests and imaging studies. RB, CA, and RS met and reviewed the labs, imaging, and WES results of the patient, and RB and RS obtained written informed consent from the parents to have their daughter's case reported. RB then invited AE-H, PB, and MH, the lead authors of the previous “foundational” studies on FBXL4 deficiency to join the authorship team. PB and MH provided us with unpublished data on their previously reported patients with the same mutation as ours. AE-H also provided us with crude data from his latest review on FBXL4 deficiency and provided us with guidance on drafting the introduction and the remainder of the case description. RB drafted the preliminary copy of the entire manuscript which was subsequently revised, edited, and approved by all authors.

### Conflict of Interest Statement

The authors declare that the research was conducted in the absence of any commercial or financial relationships that could be construed as a potential conflict of interest. The reviewer DG declared a past co-authorship with one of the authors PB to the handling editor.

## Abbreviations

mtDNA: Mitochondrial DNA
FBXL4: F-box and leucine-rich repeat 4 protein
WES: Whole-exome sequencing
CRP: C-reactive peptide.
