# Supplementary material for: FBXL4-Related Mitochondrial DNA Depletion Syndrome 13 (MTDPS13): A Case Report With a Comprehensive Mutation Review
Source: Front Genet. 2019 Feb 5;10:39. doi: 10.3389/fgene.2019.00039 (PMC6370620; doi:10.3389/fgene.2019.00039)
Supplement: Supplementary file 1 [file Table_1.DOCX]

| **Study** | **Number of patients identified by the study** |
| --- | --- |
| *Bonnen et al.* | 11 (families 1 (5), 2 (5), and 3 (1)) |
| *Gai et al.* | 8 [excluding 1 from *Shamseldin et al.*] |
| *Huemer et al.* | 17 [excluding the 3 from *Gai et al.* and 1 from *Bonnen et al.*] |
| *Baroy et al.* | 1 |
| *Shamseldin et al.* | 1 |
| *Antoun et al.* | 1 |
| *Morton et al.* | 2 |
| *Dai et al.* | 9 [excluding 1 from *Antoun et al.*] |
| *van Rij et al.* | 1 |
| *Pronicka et al.* | 3 |
| *Wortmann et al.* | 2 |
| *El-Hattab et al.* | 37 |
| *N.B. The case on seizures in a patient with FBXL4- related mitochondrial encephalomyopathy [1] discusses a patient already reported previously by Huemer et al. (Patient # 21)* | |

**Supplementary table.** List of studies reported to date on patients with *FBXL4*-related MTDPS13 with the number of patients reported by each study, excluding duplicate reporting.

1. Ebrahimi-Fakhari, D., et al., *Recurrent Stroke-Like Episodes in FBXL4-Associated Early-Onset Mitochondrial Encephalomyopathy.* Pediatr Neurol, 2015. **53**(6): p. 549-50.
